# 

**Published:** 1913-09

**Authors:** 


					CONTENTS.
Pagt
"^dicine and its Practitioners during the Earlier Years of the
kl.
History of Bath.
By James Wigmore,
193
/ - -   ? J J ? ^
nYPernephroma or Mesothelioma of the Kidney.
By James Swain,
M.S., M.D. Lond., F.R.C.S. ..
213
^ Nephropexy.
By J. Lacy Firth, M.D., M.S.Lond., F.R.C.S.
^'Xed Infections.
By I. Walker Hall, M.D.
227
_ Case of Cervical Rib.
By Rupert Waterhouse, M.D., M.R.C.P.
232
hree Cases of Ataxia.
By J. M. Fortescue-Brickdale, M.A..
M.D., B.Ch. Oxon.
235
ntermittent Swelling of the Parotid Glands.
By C. W. J. Brasher,
Tl
M.D., Ch.B.Bristol
.. 23?
Ethical Aspects of Physical Research.
By the Rev. de Lacy
O'Leary, D.D.
242
n Hemorrhagic Diseases of the New-born, with Report of a
Case due to Duodenal Ulcer.
By W. J. H. Pinniger, M.D..
D o.S.Lond., M.R.C.S., L.R.C.P.
248
r?gress of the Medical Sciences:
?vied i
By George Parker, M.A., M.D. Cantab.
256
Surgery.
By A. Rendle Short, M.D., B.S., B.Sc. Lond., F.R.C.S.
262
j3 Ophthalmology.
By Cyril H. Walker, M.B., B.A.Cantab.
265
n - fiviiaiMiwiv^^
?V|ews of Books
268
^?"apeutic Notes.
By J. M. Fortescue - Brickdale, M.A.,
M.D , B.Ch. Oxon.
279
?ies on Preparations for the Sick
282
n > ? - -r?       "* ?" "? ??? ?-
? Library of the Bristol Medico-Chirurgical Society ... 285
b'tuary Notice
287
?cal Medical Notes
287

				

